# Altered amygdala shape trajectories and emotion recognition in youth at familial high risk of schizophrenia who develop psychosis

**DOI:** 10.1038/s41398-022-01957-3

**Published:** 2022-05-13

**Authors:** Synthia Guimond, Suraj S. Mothi, Carolina Makowski, M. Mallar Chakravarty, Matcheri S. Keshavan

**Affiliations:** 1 Department of Psychiatry, Beth Israel Deaconess Medical Center, Harvard Medical School, Boston, MA USA; 2 Department of Psychiatry, The Royal’s Institute of Mental Health Research, University of Ottawa, Ottawa, ON Canada; 3 Department of Psychoeducation and Psychology, University of Quebec in Outaouais, Gatineau, QC Canada; 4 Department of Psychiatry, Massachusetts General Hospital, Boston, MA USA; 5 Cerebral Imaging Centre, Douglas Mental Health University Institute, McGill University, Montreal, QC Canada; 6 McGill Centre for Integrative Neuroscience, Montreal Neurological Institute, Montreal, QC Canada; 7 Center for Multimodal Imaging and Genetics, Department of Radiology, University of California San Diego, San Diego, United States; 8 Departments of Psychiatry and Biological and Biomedical Engineering, McGill University, Montreal, QC Canada

**Keywords:** Predictive markers, Human behaviour, Schizophrenia

## Abstract

Relatives of individuals with schizophrenia have a higher risk of developing the illness compared to the general population. Thus, youth at familial high risk (FHR) offer a unique opportunity to identify neuroimaging-based endophenotypes of psychosis. Previous studies have identified lower amygdalo-hippocampal volume in FHR, as well as lower verbal memory and emotion recognition. However, whether these phenotypes increase the risk of transition to psychosis remains unclear. To determine if individuals who develop psychosis have abnormal neurodevelopmental trajectories of the amygdala and hippocampus, we investigated longitudinal changes of these structures in a unique cohort of 82 youth FHR and 56 healthy controls during a 3-year period. Ten individuals from the FHR group converted to psychosis. Longitudinal changes were compared using linear mixed-effects models. Group differences in verbal memory and emotion recognition performance at baseline were also analyzed. Surface-based morphometry measures revealed variation in amygdalar shape (concave shape of the right dorsomedial region) in those who converted to psychosis. Significantly lower emotion recognition performance at baseline was observed in converters. Percent trial-to-trial transfer on the verbal learning task was also significantly impaired in FHR, independently of the conversion status. Our results identify abnormal shape development trajectories in the dorsomedial amygdala and lower emotion recognition abilities as phenotypes of transition to psychosis. Our findings illustrate potential markers for early identification of psychosis, aiding prevention efforts in youth at risk of schizophrenia.

## Introduction

Schizophrenia is one of the most disabling medical disorders, affecting about seven individuals per 1000 worldwide [1–3]. Because schizophrenia is a highly heritable disorder, relatives of individuals with schizophrenia have a higher risk of developing the illness compared to the general population [4, 5]. Hence, unaffected youth at familial high risk (FHR) offers a unique opportunity to examine how phenotypic brain variations and cognitive profiles can help predict the predisposition of developing the illness.

Structural magnetic resonance imaging (MRI) studies have consistently shown abnormalities in the amygdala and the hippocampus in chronic and first-episode schizophrenia [6–9], as well as in FHR [10–17]. The amygdala and the hippocampi are important brain structures for memory and emotion processing [18–21]. Lower hippocampal volumes have been related to lower verbal learning in schizophrenia [22] and dysfunction in the amygdala to lower emotion recognition [23]. However, the role of the amygdala and the hippocampus in the development of these cognitive impairments is not well understood. Furthermore, little is known about the timing of these neurobiological and cognitive phenotypes and whether they may contribute to later conversion to psychosis, especially in individuals at higher risk for psychosis.

Most studies investigating the abnormal volume of the amygdala and hippocampus as predictors of conversion to psychosis in high-risk populations were performed in adult individuals or were conducted on clinical high-risk patients [24–29]. These studies have found either smaller right hippocampus volume in converters [26, 28] or no significant differences in these brain structures between converters and non-converters [24, 25, 27, 29, 30]. Variability in clinical symptomatology, age of the sample, imaging methods, and study designs (i.e., cross-sectional and longitudinal) may have contributed to the inconsistencies reported in those studies.

One hypothesis that remains to be tested is that premorbid longitudinal changes in the amygdala and the hippocampus could be associated with conversion to psychosis. Therefore, studies with younger FHR samples and longer follow-up periods are essential in understanding the role of these brain structures in the conversion to psychosis. Moreover, subtle but meaningful changes in these brain structures may not be captured by their total volume [31]. Hence, considering the shape of these structures and their constituents (e.g., hippocampal subfields) is not only relevant in the context of normal development [32, 33], but can also provide an additional and novel perspective on the nature of brain structural alterations in the development of psychotic illness.

In the current study, we investigated longitudinal changes in the amygdala and hippocampus in 82 FHR youth followed for a period of 3 years. We compared those who later converted to psychosis (FHR+, *n* = 10) with those who did not (FHR−, *n* = 72) and 56 healthy controls. We had two objectives. First, we aimed to determine whether the amygdala and hippocampus (including subfields) have abnormal premorbid developmental trajectories by comparing FHR who later developed psychosis to those who did not. Second, we investigated whether abnormalities in emotion recognition and verbal learning at baseline were associated with later conversion to psychosis. We hypothesized that FHR+ would show abnormal longitudinal trajectories of both structures, as well as lower memory and emotion recognition performance compared to FHR- and healthy controls.

## Methods

### Participants

In this longitudinal study, participants consisted of relatives of individuals with a diagnosis of schizophrenia or schizoaffective disorder (FHR: *n* = 82; 67 first-degree relatives, 15 second-degree relatives) and 56 healthy controls with no first- or second-degree relatives with a psychotic disorder. Controls were recruited via advertisements in the same community locations as FHR participants. FHR participants were recruited by approaching patients with schizophrenia with eligible relatives in outpatient clinical services at the Western Psychiatric Institute and Clinic, Pittsburgh, or related clinical sites. The Structured Clinical Interview for DSM-IV Disorders (SCID) [34], as well as a consensus diagnosis based on all available health records and interviews with key informants (parents, guardians), were used to confirm the schizophrenia or schizoaffective diagnosis of the patients. These consensus discussions, led by Matcheri Keshavan MD and Debra Montrose PhD yielded lifetime diagnoses prior to enrollment in the study and ruled out the presence of a previous psychotic disorder in FHR and control participants. For participants under the age of 15 years old, the SCID was supplemented by the Developmental Disorders modules of the Kiddie Schedule for Affective Disorders and Schizophrenia (K-SADS) [35]. The raters interviewing FHR and healthy controls were not blind to the parental diagnoses (this was difficult, since the assessors were often involved in recruiting the participants from the clinics) (for details see ref. [36]).

Inclusion criteria for all participants were: intelligence quotient (IQ) ≥ 80 as determined by the Revised Wechsler Adult Intelligence Scale [37], no lifetime evidence of a psychotic disorder, no previous exposure to antipsychotic medications, no substance abuse within the past month or dependence upon substances within the past 6 months, no significant neurological or medical conditions, no MRI contraindications, and fluency in English. All participants received a complete explanation of the experiment and signed consent. Participants younger than 18 years of age gave informed assent and the parent or guardian signed consent. Data used in the current analyses were collected from 1995 to 2008. This research was approved by the University of Pittsburgh Medical Center’s Institutional Review Board.

**Fig. 1:**
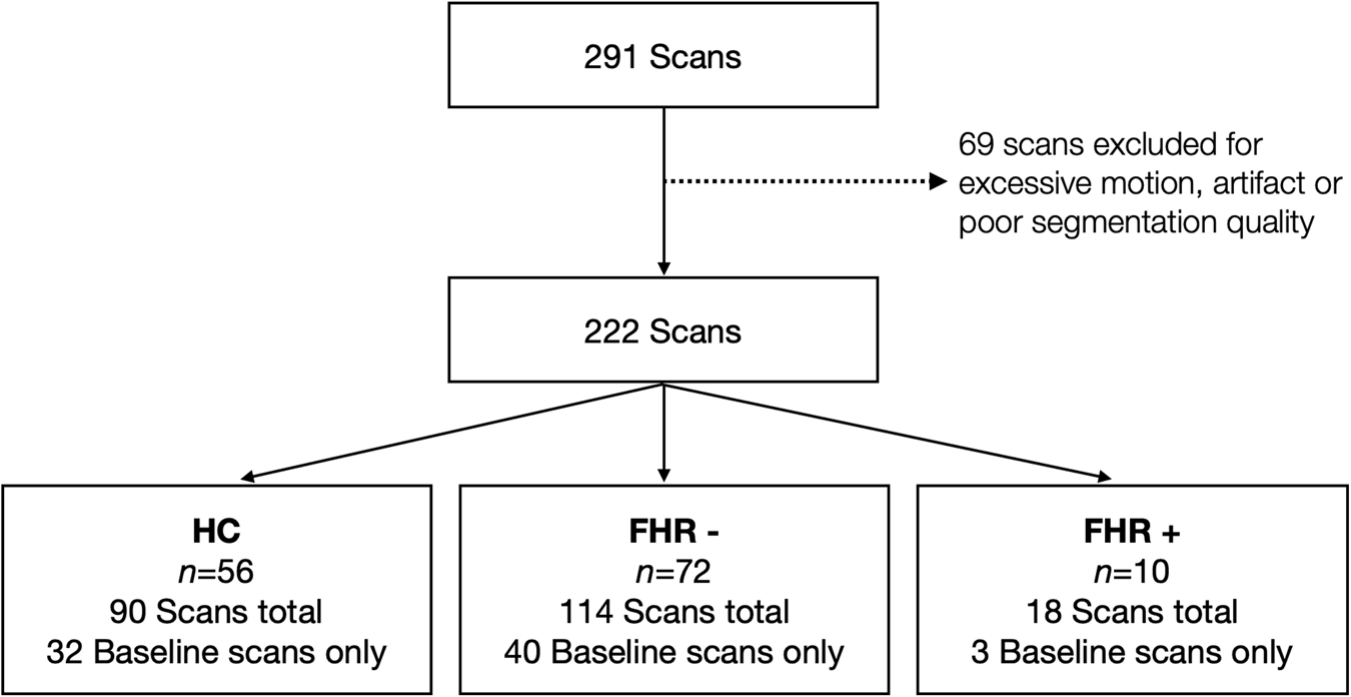
Details on scans and participants included in our analyses. From the 69 excluded scans, 4 were FHR+, 32 FHR- and 33 HC. FHR− = familial high risk who did not convert to psychosis, FHR+ = familial high risk who converted to psychosis, HC = healthy controls.

All participants were followed up at approximately annual intervals for up to 3 years. After data quality control, 75 participants only had data collected at one-time point (Fig. 1). The mean total follow-up duration for the participants with more than one-time point (*n* = 63) was 20.09 months (min = 9.19 months; max = 40.44 months), and there were no significant differences between our groups (*p* = 0.63). Conversion to psychosis was determined at follow-up visits by trained clinicians using the SCID/K-SADS, historical data acquired at baseline, subsequent evaluations, chart reviews, and collateral information from patients, families, and guardians where available. All available and relevant information was used in regular consensus diagnostic meetings, which were chaired by a senior clinician. By the end of the study, ten FHR had developed a psychotic disorder (schizophrenia (*n* = 4), schizoaffective disorder (*n* = 3), schizophreniform (*n* = 1), and psychosis not otherwise specified (*n* = 2)). Converters included six first-degree relatives and four second-degree relatives.

### MRI acquisition and preprocessing

Three-dimensional spoiled gradient recall acquisition (SPGR) T1-weighted scans were collected using a 1.5 T GE SIGNA imaging system (General Electric Healthcare, Marlborough, MA, USA) at baseline and annual intervals for up to 3 years. T1-weighted scans were acquired with 124 coronal slices, TR = 25 ms, and TE = 5 ms, 256 × 192 matrix, and slice thickness = 1.5 mm without interslice gap. Motion and field inhomogeneity artifacts were first graded (0 = no/very subtle motion, 1 = moderate motion, 2 = severe motion), and scans with moderate-to-severe motion artifacts were excluded from the study (Fig. 1). Pre-processing of T1-weighted images was carried out using the minc bpipe library (https://github.com/CobraLab/minc-bpipe-library).

### MAGeT-brain segmentation

The amygdala, hippocampus and hippocampal subfields (i.e., CA1, CA2/CA3, CA4/dentate gyrus, subiculum, and stratum) were segmented on the pre-processed T1-weighted images of all subjects [38]. Segmentations were performed using the Multiple Automatically Generated Templates (MAGeT) Brain segmentation algorithm, which leverages the neuroanatomical variability of a participant population to boost segmentation accuracy [39–41]. Each step of the MAGet-Brain segmentation is detailed in Supplementary Information. Scans were then removed from the analyses due to low segmentation quality through a visual inspection of all MAGeT segmentations using a similar grading as the quality control of motion artifacts (Fig. 1). Total brain volumes were also estimated using the CIVET pipeline (Version 2.0.0: http://www.bic.mni.mcgill.ca/ServicesSoftware/CIVET) [42].

### Morphometric modeling

To estimate surface-based deformations, a single averaged transformation was first estimated by concatenating the individual nonlinear deformations from each subject back to a model generated from the atlases. These transformations were then averaged into a single non-linear transformation for each model-to-subject pathway to reduce noise in the transformation and to increase precision and accuracy [43]. All surface area values were blurred with a surface-based diffusion smoothing kernel of 5 mm. To determine the shape difference at the corresponding vertices of an individual’s amygdala and hippocampus and the model surface, we used the dot product of the unit vector lying normal to the model surface at each vertex. Then, the vector from the final averaged non-linear deformation field at that same vertex was evaluated. This determined the magnitude of local inward/outward displacement in the direction normalized to the model surface at each vertex (i.e., concave/convex shape, see Supplementary Information).

### Cognitive measures

The California Verbal Learning Test (CVLT)’s total recall for trial 1, percent trial to trial transfer, and delayed total recall were used to assess verbal learning ability [44]. Emotion recognition was assessed using the total correct responses on the Penn Emotion Recognition Test-40, a facial emotion recognition paradigm commonly employed in schizophrenia research [45].

### Statistical analysis

All analyses were performed using SPSS (version 21) and SurfStat toolbox within Matlab (http://www.math.mcgill.ca/keith/surfstat/). The analyses were two-tailed with a critical *p*-value of 0.05, and the levels of significance were corrected in all analyses for multiple comparisons using the false discovery rate (FDR) approach. Voxel-wise FDR corrections were separately applied within each region of interest (i.e., each amygdala and hippocampus).

#### Demographic analysis

Demographic variables in Table 1 were analyzed with a one-way analysis of variance for continuous variables (i.e., age) and chi-square for categorical variables (i.e., sex, handedness, and race).

**Table 1:** Demographics of all participants.

	HC			FHR−			FHR+			Between-group comparison
	(*N* = 56)			(*N* = 72)			(*N* = 10)			
	Mean	SD	Range	Mean	SD	Range	Mean	SD	Range	*p*-value
Age	18.0	3.8	9–25	16.9	3.7	9-24	17.1	3.6	10–20	0.232
	*N*	%		*N*	%		*N*	%		
Sex										0.299
Male	20	35.7		32	44.4		6	60.0		
Female	36	64.3		40	55.6		4	40.0		
Handedness										
Right	49	87.5		59	81.9		7	70.0		0.333
Mixed	0	0.0		3	4.2		1	10.0		
Left	2	3.5		2	2.8		0	0.0		
Race										
Caucasian	43	76.8		34	47.2		3	30.0		0.006
African American	11	19.6		37	51.4		7	70.0		
Asian	1	1.8		1	1.4		0	0.0		
Other	1	1.8		0	0.0		0	0.0		

We considered the first time point with good quality data available as baseline for each participant. *P*-values reported in the table are from the omnibus tests. Handedness data were missing for 5 HC and 5 FHR. FHR− = familial high risk who did not convert to psychosis, FHR+= familial high risk who converted to psychosis, HC = healthy controls, SD = standard deviation.

#### Amygdala and hippocampus analysis

To examine longitudinal trajectories of the amygdala and the hippocampus (including subfields), we computed the slopes of change for each participant. Available timepoints were age-centered and subject-specific slopes were calculated across available timepoints [46]. Group differences and group-by-centered-age interactions (i.e., group differences in the slope of amygdalar-hippocampal changes with age) were the main predictors of interest. We used a series of linear mixed effects models, accounting for random intercept and controlling for sex, handedness, and race. Estimated total brain volume was also entered as a covariate for all imaging analyses. When appropriate, post-hoc pairwise between-group comparisons were also performed.

#### Cognitive measures analysis

We considered the first time point with good quality data available as the baseline for each participant. Cognitive measures at baseline were only available on a subsample of participants (see the Supplementary Information for demographics of those subsamples). Group differences at baseline in verbal learning and emotion recognition performance were investigated using a general linear model (GLM). Race was then entered as a covariate to ensure the effects remained significant.

## Results

### Demographics results

Table 1 provides the demographics of all participants. There were no significant group differences in age, handedness, or sex. The race was significantly different between our groups for the main imaging sample, but not for the subsamples of participants with cognitive data (see Supplementary Information).

### The amygdala and hippocampus

**Fig. 2:**
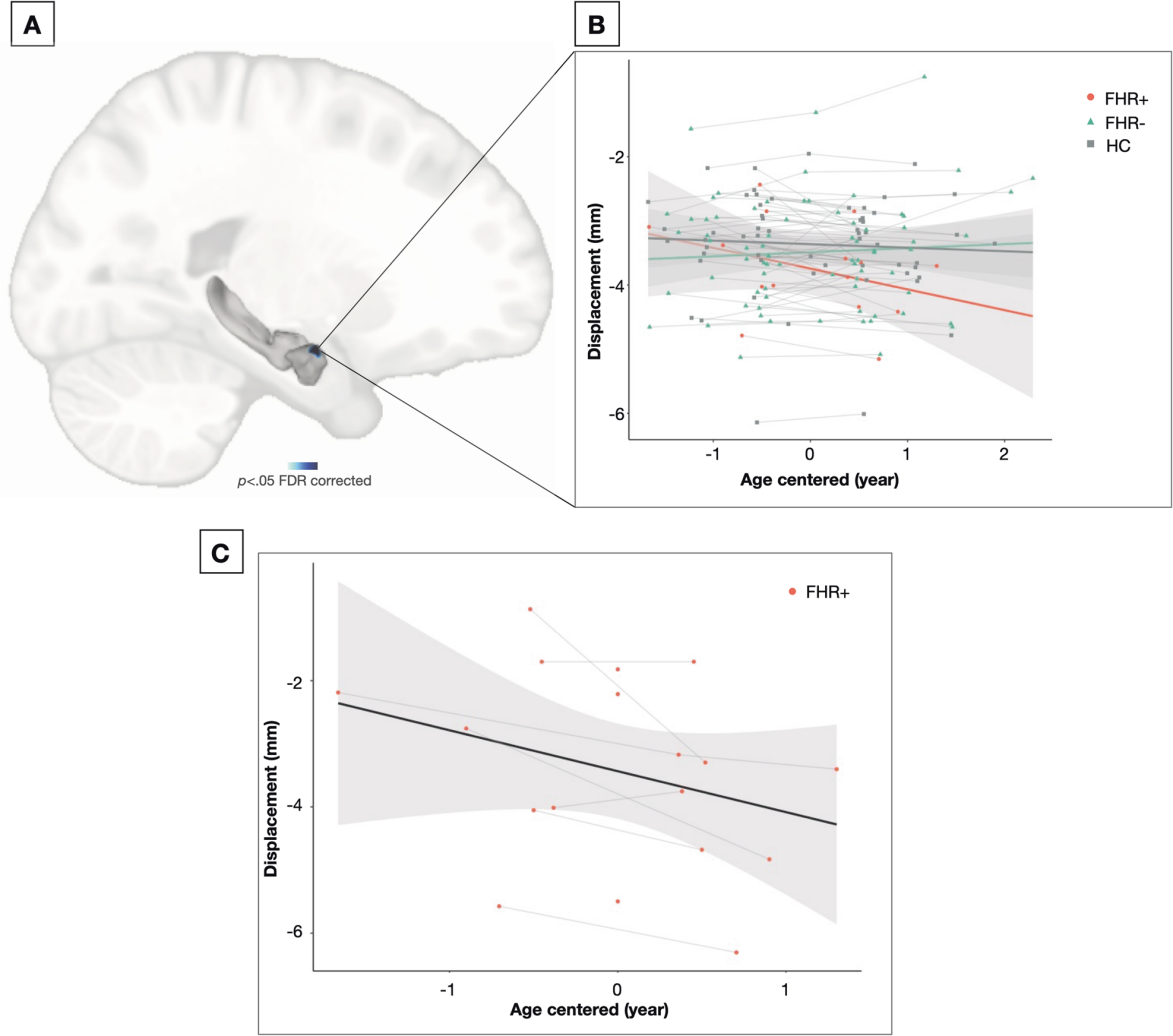
Between-group differences in amygdalar shape trajectories. **A** Significant group-by-centered-age interaction on the shape of the dorsomedial amygdala (*p* < 0.05 corrected). **B** Decreased peak displacement (or increase in concavity) over the years in FHR+ (*n* = 10) compared to FHR− (*n* = 72) and HC (*n* = 56). **C** Extent of changes in amygdala shape over time in the FHR− (*n* = 10). FHR− = familial high risk who did not convert to psychosis, FHR+ = familial high risk who converted to psychosis, HC = healthy controls.

We observed a significant group-by-centered-age interaction for the shape of the right dorsomedial amygdala (*p* < 0.05 FDR corrected) (Fig. 2A). Pairwise contrast comparisons showed that FHR+ had significantly altered shape trajectories in this region compared to FHR− (*p* < 0.05 FDR corrected). More specifically, FHR+ showed a significant decrease in displacement (more concave) with age in this region, while no change over time was observed in FHR− (Fig. 2B and C). No post-hoc pairwise interactions specifically with HC were significant (*p* > 0.05 FDR corrected).

We did not observe any statistically significant group difference nor group-by-centered-age interaction for the total volume of the amygdala and the hippocampus (Table 2).

**Table 2:** Total volume results.

	Main effect of group		Group-by-centered-age interaction	
	*F*-value	*p*-value	*F*-value	*p*-value
Amygdala				
Left	1.044	0.526	0.790	0.370
Right	0.715	0.572	0.111	0.717
Hippocampus				
Left	0.217	0.671	0.184	0.680
Right	0.272	0.814	0.670	0.830

*p*-values are uncorrected.

We found a trend-like group-by-centered-age interaction for the subiculum volume (*p* = 0.074 uncorrected). This interaction was driven by a slight increase in subiculum volume with age in controls, which was not found in FHR- (see Supplementary Information). No other shape or volume changes of the hippocampal subfields were significantly different between groups (see Supplementary Information).

### Cognitive measures

#### Emotion recognition

**Fig. 3:**
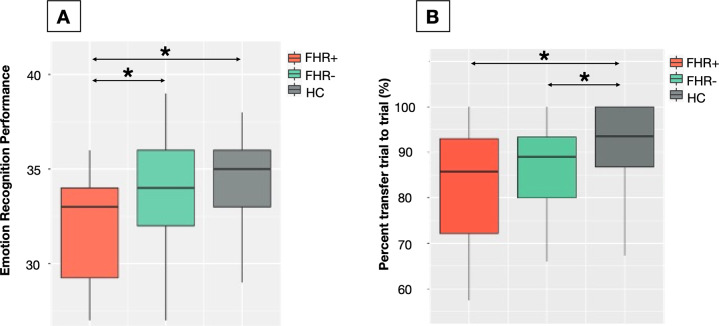
Between-group differences on emotion recognition and verbal learning performance at baseline. **A** Significantly lower emotion recognition performance at baseline in FHR+ (*n* = 8) compared to FHR- (*n* = 37) and HC (*n* = 39) (*p < 0.05 corrected). **B** Significant lower verbal learning percent transfer trial to trial at baseline in both FHR groups (FHR+ *n* = 10; FHR*− n* = 43) compared to HC (*n* = 36) (*p < 0.05 corrected). FHR− = familial high risk who did not convert to psychosis, FHR+ = familial high risk who converted to psychosis, HC = healthy controls, Plain line = FHR, Dashed line = HC.

At baseline, we observed a significant group difference on emotion recognition performance (*F*_(2,81)_ = 3.925, *p* = 0.024). This finding remained significant when controlling for race (*F*_(2,78)_ = 3.733, *p* = 0.028). More specifically, FHR+ had significantly lower emotion recognition performance compared to FHR− (*p* = 0.039), and healthy controls (*p* = 0.007) (Table 3 and Fig. 3A). The difference between FHR- and healthy controls was not significant (*p* = 0.257).

**Table 3:** Between-group differences on cognitive performance at baseline.

	HC			FHR−			FHR+					
	Mean	SD	*N*	Mean	SD	*N*	Mean	SD	*N*	*F*-value	*p*-value	Pairwise comparisons
*Emotion recognition*												
Penn Test-40	34.36	1.94	39	33.51	3.77	37	30.88	5.19	8	3.925	**0.024**	FHR+ < FHR−, FHR+ < HC
*Verbal learning*												
CVLT total trial 1 recall	10.19	2.02	37	9.94	2.58	48	8.70	3.4	10	0.367	0.694	N.S.
CVLT percent trial to trial transfer	91.53	8.62	36	85.21	14.56	44	82.32	14.25	10	4.162	0.019	FHR+ < HC, FHR− < HC
CVLT delayed correct recall	14.08	2.10	37	13.52	2.78	48	13.80	2.39	10	0.223	0.8	N.S.

*p*-values survived FDR correction for multiple comparisons (*p* < 0.05). One entry was missing for the percent to trial transfer and 4 outliers (>3 SD) for this variable were removed from the analysis. FHR− = familial high risk who did not convert to psychosis, FHR+ = familial high risk who converted to psychosis, HC = healthy controls, *N* = number of participants with data available at baseline and included in the analysis, SD = standard deviation, N.S. = not significant.

#### Verbal learning

No significant difference was observed between any groups on total recall on trial 1 (*F*_(2,90)_ = 0.367, *p* = 0.694) and delayed total recall (*F*_(2,91)_ = 0.223, *p* = 0.800). However, both FHR+ and FHR− presented significantly lower percent trial to trial transfer compared to healthy controls (see Table 3 and Fig. 3B, omnibus test: *F*_(2,86)_ = 4.162, *p* = 0.019, FHR−: *p* = 0.011, FHR+: *p* = 0.040). FHR+ and FHR− did not significantly differ on this measure (*p* = 0.198). The observed group difference in the percent trial to trial transfer was only trend-like after controlling for race (*F*_(2,83)_ = 2.276, *p* = 0.109).

## Discussion

The current study identified two markers of risk of conversion to psychosis in youth FHR: (1) abnormal changes in the shape of the amygdala and (2) lower emotion recognition ability. More specifically, FHR+ showed decreased longitudinal displacement (or increased concavity) in the right dorsomedial region of the amygdala and impaired emotion recognition performance at baseline compared to FHR−. These specific neurobiological and cognitive markers could facilitate the early identification of youth FHR that are more at risk of developing a psychotic disorder.

Our results are in line with previous reports showing that shape characteristics of a structure can provide information that is neuroanatomically unique in relation to volumetric assessment [47–49]. In the current study, we did not observe a significant longitudinal change in terms of the total volume of the amygdala between the groups, but we found a significant change in the right amygdalar shape in a dorsomedial region. Our results support those from Bois and colleagues who did not observe any significant differences in the volumes of the amygdala or hippocampus between FHR converters and non-converters [24, 25]. These findings vary from previous reports using voxel-based morphometry and showing change over time in gray matter in or around the hippocampus in high-risk individuals who convert to psychosis [50, 51]. Variabilities in previous findings could be explained by differences in imaging methods used to assess changes in limbic structures (i.e., voxel-based morphometry versus automated volume segmentation). Our current findings further suggest that the shape of a subcortical structure, like the amygdala, maybe a more sensitive measure than its volume to identify biomarkers of risk of conversion to psychosis.

The amygdala plays a key role in emotion recognition [18, 19]. The ability to distinguish different emotions in people is essential for our social interactions. This specific ability is impaired in schizophrenia [52, 53] and similar difficulties have been observed in people at clinical high risk [54], as well as in FHR [55, 56]. Interestingly, lower emotion recognition in clinical high risk for schizophrenia has been previously associated with conversion to psychosis [57]. Our current findings support these previous reports and provide additional evidence for an association between diminished emotion recognition capacity in FHR and later conversion to psychosis. Our results also highlight the potential specific role of the medial amygdala in emotion processing [58, 59]. Longitudinal changes in medial amygdalar shape have also been recently observed in first-episode psychosis, specifically in patients with persistent negative symptoms [60]. In future studies, it would be of interest to explore links between impaired emotion processing in earlier stages of psychosis and markers of amygdalar shape by incorporating other relevant measures, such as genetic information and negative symptoms.

In addition to its role in processing emotions, the medial amygdala is also important for reward and stress responses. For example, reduced attention to rewarding stimuli has been previously associated with hypofunctionality of the centromedial nucleus of the amygdala in FHR [61]. Neurons in the medial region of the amygdala have also shown reduced spine density after chronic stress exposure in mice [62]. Given the role of the medial amygdala in response to emotions, rewarding stimuli, and stress, it is possible that early morphometric abnormalities that we observed in this region could be associated with greater stress vulnerability. One hypothesis that remains to be tested is that lower emotion processing abilities in FHR and reduced attention to rewarding stimuli could increase vulnerability to stress and risk of conversion to psychosis. While our current results support the role of lower emotion processing and abnormal development of the dorsomedial amygdala in conversion to psychosis for FHR, future studies are needed to investigate potential mediators of reward and stress response.

Contrary to our initial hypothesis, we did not observe any group differences in total volume or shape of the hippocampus, but we observed a trend-like group-by-centered-age interaction for the subiculum volume between FHR- and controls. The subiculum is an important region of the hippocampus implicated in learning and memory [63, 64]. Previous studies also observed smaller hippocampus and subiculum volumes in adult FHR [25, 65]. Our current results in youth FHR were only trend-like significant for the subiculum; it is possible that such abnormalities could be more predominant after adolescence. However, more longitudinal studies in FHR with larger samples are needed to draw firmer conclusions.

The current study has many strengths. First, we investigated a unique sample of youth and antipsychotic-free FHR, using both cognitive and brain imaging data. Second, neuroimaging data were collected at many time points during a follow-up period of 3 years. Third, in addition to total volumetric analyses, we used subfield volumetric analyses and shape analysis methodology to examine longitudinal changes in these brain regions. Together, this allowed us to identify more subtle but meaningful brain differences between converters and non-converters.

Nonetheless, our findings should be interpreted in light of some limitations. For instance, cognitive measures were only collected on a subsample of our participants. Longitudinal imaging studies investigating youth over a long period of time and with many timepoints are challenging. In addition, only 56 of our participants had longitudinal imaging data that could be used in our analyses. While our sample size and follow-up attrition are in the range of similar longitudinal investigations previously published [25], we did not have sufficient power to use predictive statistical models to determine whether the shape abnormality or lower emotion recognition performance were predictive of conversion to psychosis. Furthermore, while MAGeT Brain has been extensively validated on data acquired on a 1.5-T scanner [41, 60], the scanner resolution could have limited the precision of segmentation for the hippocampal subfields which might have increased the risk of false-negative findings in the current study. Longitudinal studies with larger samples of youth FHR and higher scanner resolution are needed and encouraged.

## Conclusion

Youth FHR who developed a psychotic disorder had significantly lower emotion recognition performance at baseline and abnormal age-related shape trajectories in the right dorsomedial amygdala compared to other FHR individuals. These findings could improve the early detection of youth who are at higher risk of developing psychosis. They could also guide the development of future preventive intervention on specific cognitive and neurobiological targets.

## Supplementary information

Supplementary Information
