# Supplementary material for: Altered amygdala shape trajectories and emotion recognition in youth at familial high risk of schizophrenia who develop psychosis
Source: Transl Psychiatry. 2022 May 13;12:202. doi: 10.1038/s41398-022-01957-3 (PMC9106712; doi:10.1038/s41398-022-01957-3)
Supplement: Supplementary file 1 — Supplementary Information [file 41398_2022_1957_MOESM1_ESM.pdf]

Supplementary Information

1. Supplementary methods

MAGeT-Brain was configured using a template library composed of 21 images selected from the unlabeled participants that were representative of the age, sex and race range of the entire sample. Each atlas label was first propagated to label each template image using non-linear transformation estimates that match each atlas to each template. Registration was carried out using the Advanced Normalization Tools (ANTs; https://github.com/vfonov) (Avants, et al., 2009) for MINC formatted images (McConnell Brain Imaging Centre, Montreal Neurological Institute, McGill University; http://bic-mni.github.io).

Based on a five-image atlas library, each template image received five separate labels (one from each atlas). Labels from each template image were then similarly propagated to each unlabeled subject image, resulting in 100 candidate labels for each subject. To limit errors due to resampling or registration, voxel-wise majority voting was used to fuse the candidate labels for each participant into a single consensus label (Park, et al., 2014; Pipitone, et al., 2014).

Segmented images were then inspected by one of the authors (SG) to ensure high segmentation quality (see example of segmentation on Supplementary Figure 1).

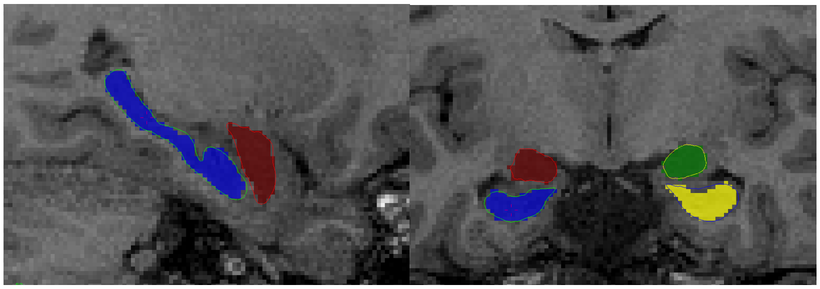

Supplementary Figure 1. (Modified with permission from Supplementary material of Makowski et al. (2017)) Example of segmentation of bilateral hippocampus and amygdala from the MAGeT-Brain algorithm. Blue = Left hippocampus, Yellow = Right hippocampus, Red = Left amygdala, Green = Right amygdala.

Motion can have significant impact on downstream results, especially when comparing patients to healthy controls and when working with adolescent population (Makowski, et al. 2019; Pardoe, et al. 2016; Weinberger & Radulescu 2016). Therefore, a stringent quality control procedure was used to ensure only high quality data was retained in the analyses. A total of 223 high quality scans were included in the analyses. From these 223 scans, 76 baseline scans did not have any follow-up scan (attrition of 34%).

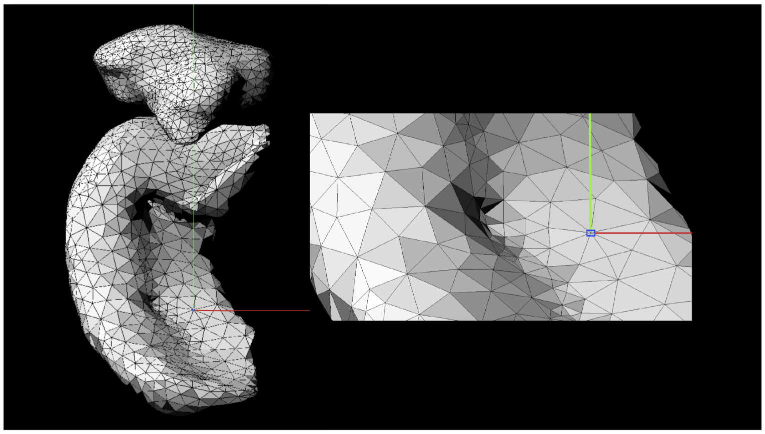

Supplementary Figure 2. (Reproduced with permission from the Supplementary material of Makowski, et al. 2017) Three-dimensional mesh overlaid on a model surface of the amygdala and hippocampus used for performing the shape analysis. The left panel presents the dorsal view and the right panel shows the intersection between vertices used to measure displacement compare to the model (i.e., concave or convex shape).

2. Supplementary results

We did not observe any significant difference in terms of age, sex or race in any of the subsamples used for the cognitive analyses (Supplementary Table 1 and 2).

Supplementary Table 1. Demographics of participants with emotion recognition data

Variable	HC (*n* = 39)			FHR – (*n* = 37)			FHR + (*n* = 8)			Between-group comparison
	Mean	*SD*	*Range*	*Mean*	*SD*	*Range*	*Mean*	*SD*	*Range*	*p*
Age	17.6	3.1	16-18	17.3	3.4	16-18	17.9	3.1	15-20	.886
	N	%		N	%		N	%		
Sex										
Male	14	35.9		16	43.2		6	75		.126
Female	25	64.1		21	56.8		2	25		
Race										
Caucasian	28	71.8		22	59.5		3	37.5		.288
African American	9	23		15	40.5		5	62.5		
Asian	1	2.6		0	0		0	0.0		
Other	1	2.6		0	0.0		0	0.0		

Supplementary Table 2. Demographics of participants with verbal memory percent trial to trial data

Variable	HC (*n* = 36)			FHR – (*n* = 43)			FHR + (*n* = 10)			Between-group comparison
	M	*SD*	*Range*	*M*	*SD*	*Range*	*M*	*SD*	*Range*	*p*
Age	17.9	2.9	17-19	16.9	3.6	16-18	17.1	3.6	15-20	.443

	N	%	N	%	N	%	
				Sex			
Male	12	33.3	16	37.2	6	60	.302
Female	24	66.7	27	62.8	4	40	
				Race			
Caucasian	27	75	23	53.5	3	30	.076
African American	7	19.4	19	44.2	7	70	
Asian	1	2.8	1	2.3	0	0.0	
Other	1	2.8	0	0.0	0	0.0	

We did not observe any significant main effect of group nor centered-age-by-group interaction for the hippocampus subfields (Supplementary Table 3).

Supplementary Table 3. Hippocampus subfields results

	Main effect of group		Centered-age-by-group interaction	
**Subfields**	*F*	*p*	*F*	*p*
Right CA1	0.030	.489	1.120	.280
Right subiculum	1.392	.462	0.220	.770
Right CA4/dentate gyrus	0.917	.912	0.308	.233
Right CA2/CA3	0.150	.566	0.360	.69
Right stratum	0.340	.600	0.450	.861
Left CA1	0.223	.474	0.921	.947
Left subiculum	0.583	.480	0.925	**.074**
Left CA4/dentate gyrus	1.480	.973	0.844	.412
Left CA2/CA3	1.108	.950	1.033	.853
Left stratum	0.067	.6724	0.459	.887

Note: Bold *p*-value represents uncorrected trend-like significant result (*p* <.01).

We only observed a trend-like centered-age-by-group interaction on the left subiculum. Post-hoc pairwise comparison showed that this trend-like interaction was driven by a slight increase in the left subiculum volume over time in HC compared to FHR- (F = 2.120, p = .074). While the FHR+ appear to have overall lower left subiculum volume compared to HC and FHR-, this difference was not significant (F = 0.520, p = .230).

Supplementary references

Avants, B. B., Tustison, N., & Song, G. (2009). Advanced normalization tools (ANTS). *Insight j*, *2*, 1-35.

Makowski, C., Bodnar, M., Shenker, J. J., Malla, A. K., Joober, R., Chakravarty, M. M., & Lepage, M. (2017). Linking persistent negative symptoms to amygdala–hippocampus structure in first-episode psychosis. *Translational psychiatry*, *7*(8), e1195.

Makowski, C., Lepage, M., & Evans, A. C. (2019). Head motion: the dirty little secret of neuroimaging in psychiatry. *Journal of psychiatry & neuroscience: JPN*, *44*(1), 62.

Pardoe, H. R., Hiess, R. K., & Kuzniecky, R. (2016). Motion and morphometry in clinical and nonclinical populations. *Neuroimage*, *135*, 177-185.

Park, M. T. M., Pipitone, J., Baer, L. H., Winterburn, J. L., Shah, Y., Chavez, S., ... & Chakravarty, M. M. (2014). Derivation of high-resolution MRI atlases of the human cerebellum at 3 T and segmentation using multiple automatically generated templates. *Neuroimage*, *95*, 217-231.

Pipitone, J., Park, M. T. M., Winterburn, J., Lett, T. A., Lerch, J. P., Pruessner, J. C., ... & Alzheimer's Disease Neuroimaging Initiative. (2014). Multi-atlas segmentation of the whole hippocampus and subfields using multiple automatically generated templates. *Neuroimage*, *101*, 494-512.

Weinberger, D. R., & Radulescu, E. (2016). Finding the elusive psychiatric “lesion” with 21st-century neuroanatomy: a note of caution. *American Journal of Psychiatry*, *173*(1), 27-33.
